# Inhibition of Cell Growth and Cellular Protein, DNA and RNA Synthesis in Human Hepatoma (HepG2) Cells by Ethanol Extract of Abnormal Savda Munziq of Traditional Uighur Medicine

**DOI:** 10.1093/ecam/nen062

**Published:** 2011-06-18

**Authors:** Halmurat Upur, Abdiryim Yusup, Isabelle Baudrimont, Anwar Umar, Benedicte Berke, Dilxat Yimit, Jaya Conser Lapham, Edmon E. Creppy, Nicholas Moore

**Affiliations:** ^1^Faculty of Traditional Uighur Medicine, Xinjiang Medical University, 830011 Urumqi, Xinjiang, China; ^2^Department of Pharmacognosy, University Victor Segalen Bordeaux 2, 33076 Bordeaux, France; ^3^Department of Toxicology, University Victor Segalen Bordeaux 2, 33076 Bordeaux, France; ^4^Department of Pharmacology, University Victor Segalen Bordeaux 2, 33076 Bordeaux, France

## Abstract

Abnormal Savda Munziq (ASMq) is a traditional Uighur medicinal herbal preparation, commonly used for the treatment and prevention of cancer. We tested the effects of ethanol extract of ASMq on cultured human hepatoma cells (HepG2) to explore the mechanism of its putative anticancer properties, using the 3-(4,5-dimethylthiazol-2-yl)-2,5-diphenyltetrazolium (MTT) bromide, neutral red and lactate dehydrogenase (LDH) leakage assays, testing the incorporation of ^3^[H]-leucine and ^3^[H]-nucleosides into protein, DNA and RNA, and quantifying the formation of malondialdehyde-thiobarbituric acid (MDA) adducts. ASMq ethanol extract significantly inhibited the growth of HepG2 and cell viability, increased the leakage of LDH after 48 hours or 72 hours treatment, in a concentration- and time-dependent manner (*P* < .05). Cellular protein, DNA and RNA synthesis were inhibited in a concentration- and time-dependent manner (*P* < .05). No significant MDA release in culture medium and no lipid peroxidation in cells were observed. The results suggest that the cytotoxic effects of ASMq ethanol extract might be related to inhibition of cancer cell growth, alteration of cell membrane integrity and inhibition of cellular protein, DNA and RNA synthesis.

## 1. Introduction

The therapeutic utilization of plants is part of universal human culture, and products derived from plants are frequently used for the treatment or prevention of diseases. Plants, vegetables, herbs and spices used in folk and traditional medicine are one of the sources of cancer drug discovery and development [1]. A major group of these products include pigments, vitamins, phenolic lactones, flavonoids, tannins and alkaloids [2, 3]. Although the mechanism underlying their anticancer effects is often still unclear, the fact that the consumption of fruits, vegetables and some traditional herbal preparations could lower the incidence of carcinogenesis at a wide variety of sites is broadly supported [4, 5]. The vinca alkaloids and taxane diterpenes are examples of successful plant-derived anticancer agents [6]. In the last few years, several other herbal products capable of anticancer effects have been identified, the anticancer properties of which are related to the regulation of cancer-related gene express, induction of apoptosis, cell cycle arrest and/or DNA fragmentation as well as inhibition of different cellular enzymes [7–11].

Traditional Uighur medicine, one of the main medical system in Central Asia, is based on four humors: fire, air, water and earth, which generate four body fluids: Kan (blood), Belghem (phlegm), Sapra (yellow bile) and Savda (black bile). Unbalanced body fluids will cause diseases, and traditional Uighur formulations will regulate the balance of body fluids and cure the disease [12]. Abnormal Savda Munziq (ASMq), a herbal formulation of traditional Uighur medicine, is widely used in the Xinjiang region of China, and especially for the treatment and prevention of cancer, diabetes, cardiovascular disorders as well as chronic asthma. Its anticancer properties are usually applied by Uighur physicians to the treatment and prevention of digestive cancers. For liver, stomach and colon cancers, the recommended dose is 500 mL three times a day. The putative mechanism of the anticancer effect is still unclear and needs further investigation. In a previous study, we found that ASMq aqueous extract had scavenging effects to various reactive oxygen species (ROS), and could protect against OH-induced mitochondrial and DNA oxidative damage *in vitro* [13–16].

In this article, we carried out experimental studies on ASMq ethanol extract on cultured HepG2 cells to elucidate the initial molecular mechanisms responsible for its anticancer effects.

## 2. Methods

### 2.1. Chemicals Reagents

Dulbecco's modified Eagle medium (DMEM), fetal calf serum (FCS), ethylene diamine tetra-acetic acid (EDTA), phosphate-buffered saline (PBS), *N*-lauroyl sarcosine (*N*-LS), ribonuclease A (RNase A), proteinase K, ethidium bromide, trypsine-0.02% EDTA mixture were from Sigma-Aldrich (Lyon, France). ^3^[H]-leucine, ^3^[H]-thymidine and ^3^[H]-uridine were from Perkin Elmer Life Sciences Inc. (Boston, MA, USA). All other chemicals used were of analytical grade.

### 2.2. Preparation of Ethanol Extract of ASMq

ASMq consists of powdered material of the 10 plants described in Table 1 [17]. The formulation is prepared according to a proprietary preparation [18]. A voucher of each plant is stored in the herbarium of Xinjiang Institute of Ecology and Geography, Chinese Academy of Science. Plant materials were purchased from Xinjiang Hospital of Traditional Uighur Medicine.

Powdered material (7.8 kg) was macerated in ethanol 95% [1 : 10 (w/v)] at room temperature for 4 hours with continuous stirring. The maceration was repeated three times (4 hours). After evaporation, the residue was extracted by petroleum ether. The remaining water fraction was concentrated to dryness under reduced pressure and low temperature on a rotary evaporator. Extract yield of plant material was 935 g (12%) (w/w). Extract was dissolved in distilled water (100 mg mL^−1^) and kept at −20°C prior to utilization. The other concentrations used in the experiment were prepared by addition of cell culture medium (DMEM).

### 2.3. Cell Lines and Culture Medium

Human hepatoma adherent cell lines (HepG2) were obtained from the American Type Culture Collection (ATCC). Cells were cultured and maintained with DMEM supplemented with 10% FCS, 2% l-glutamine (200 mM) and 1% penicillin-streptomycin (100 U to 100 mg mL^−1^) in a humidified 5% CO_2_–95% air mixture at 37°C.

### 2.4. MTT Assay

Cell viability was determined using MTT [3-(4,5-dimethylthiazol-2-yl)-2,5-diphenyltetrazolium bromide] assay. Cells were seeded in 96-well microplates (5000 cells/well/200 mL) and routinely cultured in a humidified incubator for 24 hours. Cell culture mediums were removed with a pipette and herbal extracts were added in serial concentrations (ranging from 0.05 to 10 mg mL^−1^) and re-incubated for 72 hours. We also tested a control group (DMEM without herbal extract), a blank group (without cells or medium). The medium with or without herbal extract was then discarded, and 100 mL of MTT solution (0.5 mg mL^−1^ in DMEM) was added to every well and re-incubated for an additional 2 hours. One hundred microliters of 10% SDS, 0.01 M HCl solution was added to each well to dissolve the formazon crystals formed. The plates were then read on a microplate reader (DYNATECH MR 4000, France) at 560 nm. In this test, four wells were used for each concentration of ASMq, and five independent experiments were performed.

### 2.5. Neutral Red Uptake Assay

Cells were seeded in 96-well microplates (10 000 cells/well/200 mL), routinely cultured in a humidified incubator for 24 hours. Cells were maintained in culture and exposed to ASMq ethanol extract over a range of concentrations (0.5–7.5 mg mL^−1^). After 48 hours exposure to ASMq, neutral red (NR) uptake test was performed according to the procedure described by Creppy [19]. Briefly, at the end of the treatment, the medium with or without ASMq was discarded and 200 *μ*L of freshly prepared neutral red solution (50 *μ*g mL^−1^) was added to every well and re-incubated for an additional 4 hours at 37°C. Thereafter, the cells were carefully washed twice with 200 *μ*L of PBS to eliminate extracellular NR. The incorporated dye was eluted from the cells by adding 200 *μ*L elution medium (50% ethanol supplemented with 1% acetic acid, v/v) into each well followed by gentle shaking of microplate for 15 min. The plates were then read at 540 nm using a microplate reader (DYNATECH MR 4000, France). The number of cells in the presence of ASMq was compared to that observed in control cultures and the percentage of viable cells calculated. The IC_50_ is determined and expressed as microgram per milliliter.

### 2.6. Lactate Dehydrogenase Leakage Assay

HepG2 cells (1 × 10^5^ cells mL^−1^well^−1^) were pre-incubated in 24-well multidishes for 24 hours at 5% CO_2_–95% air 37°C. Cell viability was assessed by lactate dehydrogenase (LDH) leakage trough the membrane into the medium. Aliquots of the cell culture supernatants from control and serial concentration of ASMq ethanol extract treated cultures were tested after 48 or 72 hours incubation for the presence of LDH using a LDH assay kit (Biomerieux, France). In this test, three wells were used for each concentration of ASMq, and three independent experiments were performed.

### 2.7. Cellular Protein, DNA and RNA Synthesis Assay

HepG2 cells (2.5 × 10^5^ cells/mL/well) were cultured in 24-well multidishes for 48 hours at 37°C. Five concentrations ranging from 0.5 to 7.5 mg mL^−1^ of extract were used. The control cultures were prepared by adding DMEM without any addition. Four wells were used for each concentration, and two independent experiments were performed. After 24 or 48 hours incubation at 37°C with or without extract, the medium removed and 2 mCi mL^−1^ of a radioactive protein synthesis precursor, ^3^[H]-leucine (specific activity, 185 GBq mmol^−1^) or 2 mCi mL^−1^ of radioactive DNA synthesis precursor, ^3^[H]-thymidine (specific activity, 3259.76 GBq mmol^−1^) or 2 mCi mL^−1^ of radioactive RNA synthesis precursor, ^3^[H]-uridine (specific activity, 888 GBq mmol^−1^) was incorporated during a 2.5 hours pulse. Once the pulse was finished, the medium was removed and cells were harvested by trypsinization. After centrifuged at 600 g for 5 min at 10°C, the supernatants were removed and cell pellets were resuspended by adding 300 mL of 20% NaOH and sonicated for 30 s with Bioblock sonicater at 75 mV, and all the samples were incubated for 20 min at 37°C. Twenty microliters of each homogenate was taken for total protein quantification using the Bradford method [20]. The samples were kept in −20°C freezer after addition of 1 mL of cold TCA (40%) until filtration. Lastly, the samples were filtered on 3 mm Whatman microfiber filter discs, which were dried at 80°C for 20 min. The radioactivity on the paper filters was counted with liquid scintillation analyzer after addition of 5 mL of Beckman Ready Organic supplemented by 0.9% acetic acid. The c.p.m. values were adjusted with total protein content in each sample. In this test, four wells were used for each concentration of ASMq ethanol extract, and three independent experiments were performed.

### 2.8. Extraction and Determination of Malondialdehyde-Thiobarbituric Acid Adduct

HepG2 (1 × 10^5^ cells/well/mL) were cultured in 24-well multidishes for 24 hours at 37°C. Five concentrations ranging from 0.5 to 7.5 mg mL^−1^ of ASMq were used. The control cultures were prepared by adding DMEM without any addition. After incubation with or without extract, the culture medium was removed. After rinsing with 0.5 mL of fresh-cold PBS twice, cells were collected by trypsinization, centrifuged at 600 g for 5 min. The cell pellets were resuspended in 270 *μ*L of STE (0.1 M NaCl, 20 mM EDTA, 50 mM Tris–HCl, pH 8.0), after adding 25 *μ*L of 7% SDS the cells were sonicated for 30s at 75 mV, 20 mL of each homogenate was taken for total protein quantification [20]. The cells were lysed with 20 *μ*L of SDS 7% (w/v), 300 *μ*L of 0.1 M HCl, 40 *μ*L of phosphotungstic acid 1% (w/v) and 300 *μ*L of 0.67% (w/v) thiobarbituric acid. The tubes were shaken for 1 min and incubated at 80°C for 1 hour in the dark. They were further placed for 20 min in an ice bath (0°C). After this period, 300 *μ*L of *n*-butanol was added and tubes were shaken vigorously for 1 min. After centrifugation for 10 min at 900 g at 4°C, the *n*-butanol phase from each sample containing MDA-TBA adduct was separated and analyzed by high performance liquid chromatography (HPLC) with fluorometric detection [21].

The HPLC system consisted of a chromatography pump (Bischoff Chromatography, Germany) equipped with a Shimadzu 8450 Fluorescence HPLC Monitor (Japan Spectroscopic Co.). Analysis of the malondialdehyde-thiobarbituric (MDA-TBA) adducts was performed at room temperature on a Ultrasep C18 column using methanol–water 40 : 60 (v/v) as the mobile phase adjusted to pH 8.3 by addition of 1 M KOH. The flow rate was maintained at 0.5 mL/min. The excitation and emission wavelengths were 515 and 553 nm, respectively. The amount of MDA-TBA measured was referred to the protein content of cellular homogenates using the Bradford method [20]. In this test, three wells were used for each concentration of ASMq, and two independent experiments were performed. Pic3 software was used for the analysis of chromatography.

### 2.9. Statistical Analysis

The data are expressed as mean ± SD of at least three independent determinations in triplicate or quadruplicate for each experimental point. The statistical differences between treated groups and control groups were determined by Student's *t*-test, and the significance threshold was set to *P* < .05.

## 3. Results

### 3.1. Inhibition of HepG2 Cell Growth by ASMq Ethanol Extract

**Figure 1:**
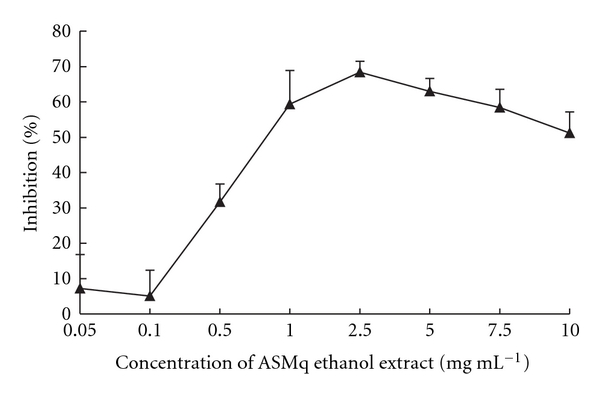
Inhibition of HepG2 cell growth by ASMq ethanol extract. Cells were treated with different concentration of ASMq ethanol extract (0.05–10 mg mL^−1^) at 37°C, 5% CO_2_ for 72 hours. Cell growth was determined by the MTT assay. Results are expressed as mean ± SD of five independent experiments.

After 72 hours incubation, ASMq ethanol extract inhibited HepG2 cell growth in a concentration-dependent manner (Figure 1). ASMq ethanol extract reached its maximum inhibitory effect (68.4%) at 2.5 mg mL^−1^ (Figure 1). The IC_50_ was determined to be 0.8 ± 0.12 mg mL^−1^.

### 3.2. Inhibition of HepG2 Cell Viability by ASMq Ethanol Extract

**Figure 2:**
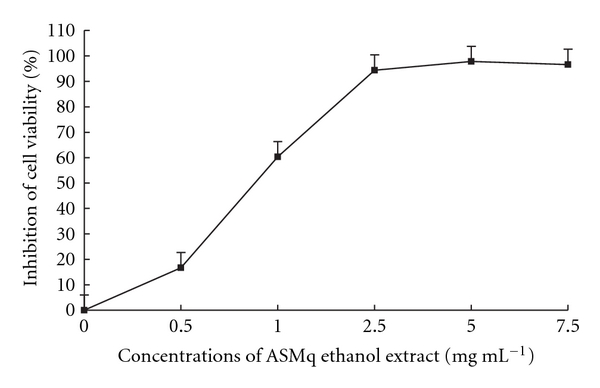
HepG2 cell viability in the presence of increasing concentrations of ASMq ethanol extract. Cells were incubated with different concentrations of ASMq aqueous extract (0.5–7.5 mg mL^−1^) at 37°C, 5%CO_2_ for 48 hours. Cell viability was determined by the neutral red test. Results are expressed as the mean ± SD of three independent experiments.

ASMq ethanol extract significantly inhibited HepG2 cell viability in a concentration-dependent manner after 48 hours incubation. IC_50_ was 0.9 ± 0.2 mg mL^−1^ (Figure 2).

### 3.3. Increase of LDH Leakage by ASMq Ethanol Extract

**Figure 3:**
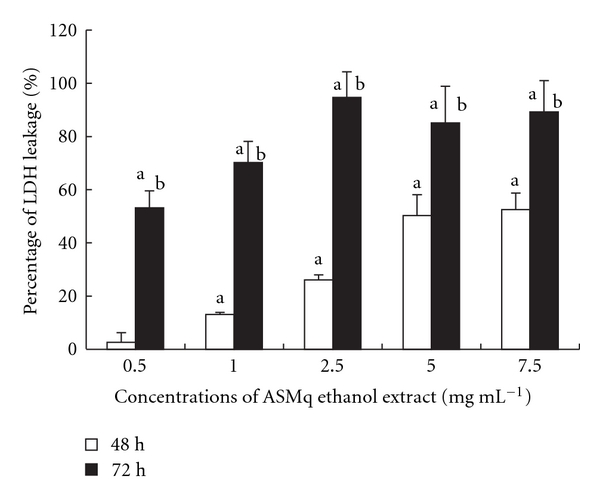
Percentage of LDH leakage into the cell culture medium after incubation of HepG2 cells with ASMq ethanol extract. Results are expressed as mean ± SD of three independent experiments.  ^a^ *P* < .05 compared with control cultures;  ^b^ *P* < .05 compared with 48 hours incubation.

There was a significant increase of HepG2 cell membrane LDH leakage after incubation with serial concentrations of ASMq ethanol extract (Figure 3). In agreement with the results of the MTT test and neutral red test, significant toxicity was observed in HepG2 cells with ASMq concentrations from 0.5 to 7.5 mg mL^−1^ after 48 or 72 hours of treatment (*P* < .05). Concentration dependence was observed at both 48 hours and 72 hours incubation. In addition, ASMq ethanol extract showed a time-dependent toxicity in our experimental condition with effects greater at 72 hours than at 48 hours (Figure 3).

### 3.4. Inhibition of Cellular Synthesis of Protein, DNA and RNA by ASMq Ethanol Extract

**Figure 4:**
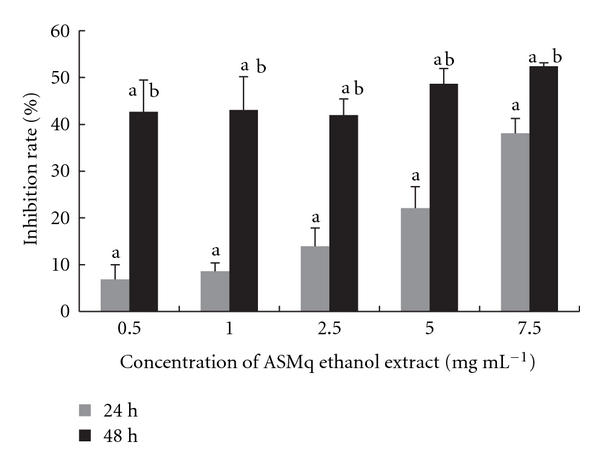
Inhibition of protein synthesis in HepG2 cells after incubation with ASMq ethanol extract. Protein synthesis was evaluated by incorporation of ^3^[H]-leucine during a 2.5 hours pulse. Results are expressed as the mean ± SD of three independent experiments.  ^a^ *P* < .05 compared with control culture;  ^b^ *P* < .05, 48 hours versus 24 hours treatment with ASMq ethanol extract.

There was a concentration-dependent inhibition of protein synthesis after 24 hours incubation. After 48 hours, the inhibition no longer was dose-dependent over the range of doses tested, but seemed stable around 43–52%. Inhibition was significantly greater at 48 than at 24 hours for all concentrations (*P* < .05) (Figure 4).

**Figure 5:**
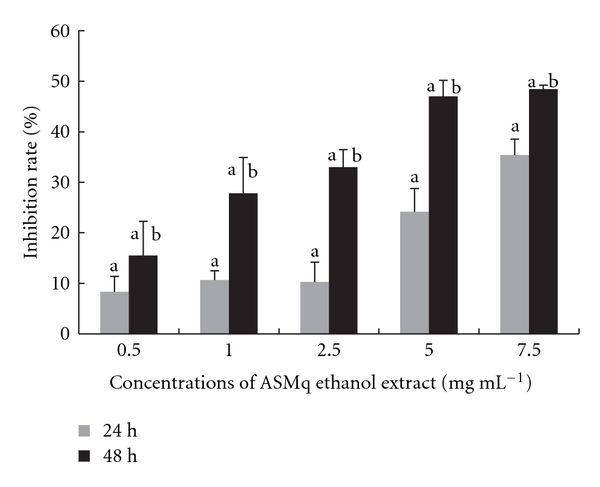
Inhibition of DNA synthesis in HepG2 cells after incubation with ASMq ethanol extract. DNA synthesis was evaluated by incorporation of ^3^[H]-thymidine during a 2.5 hours pulse. Results are expressed as the mean ± SD of three independent experiments.  ^a^ *P* < .05 compared with control culture;  ^b^ *P* < .05, 48 hours versus 24 hours treatment with ASMq.

Increasing concentrations of ASMq ethanol extract from 0.5 to 7.5 mg/mL dose-dependently inhibited cellular DNA synthesis from 8.3 to 35.4% at 24 hours, from 15.5% to 48.5% at 48 hours (Figure 5). Inhibition was greater after 48 hours than after 24 hours incubation. 

**Figure 6:**
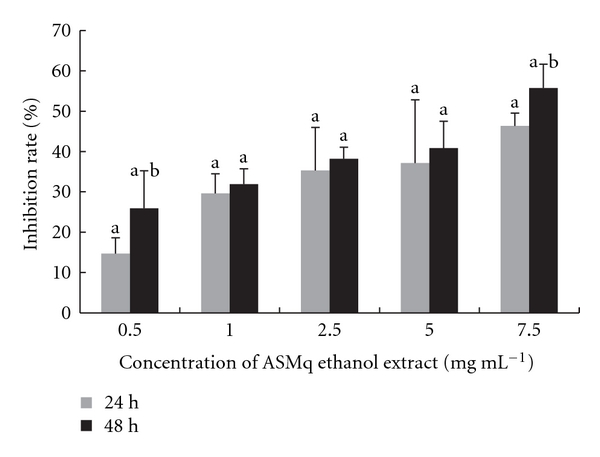
Inhibition of RNA synthesis in HepG2 cells after incubation with ASMq ethanol extract. RNA synthesis was evaluated by incorporation of ^3^[H]-uridine during a 2.5 hours pulse. Results were expressed as the mean ± SD from three independent experiments.  ^a^ *P* < .05, compared with control culture;  ^b^ *P* < .05 compared with 24 hours treatment of ASMq.

Synthesis of RNA in HepG2 cells was inhibited in a concentration-dependent manner from 14.7% to 46.4% and 26.0% to 55.7% at 24 hours and 48 hours, respectively (Figure 6). There was no clear time-dependent increase in this inhibition. 

### 3.5. Lipid Peroxidation of HepG2 Cells by ASMq Ethanol Extract

There was no significant MDA release in culture medium and no lipid peroxidation compared to controls, even at the highest concentration (10, 20 or 50 mg mL^−1^) (data not shown). This result is in accordance with a previous study showing ASMq had scavenging effects on reactive oxygen species [14].

## 4. Discussion

ASMq, used for the treatment and prevention of cancers, is made from several plants (Table 1). Pharmacological information that can be related to cancer inhibiting effects of some of the plants is outlined in Table 2.

Various antioxidative activities involving detoxification such as superoxide anion radical scavenging, inhibition of hydrogen peroxide, enhancement of superoxide dismutase, catalase and glutathion peroxidase as well as iron-chelating activities have been reported for several plants extracts such as *Anchusa italica*, *Foeniculum vulgare*, *Glycyrrhiza sp*., *Lavandula angustifolia* and *Melissa officinalis*.

Anti-inflammatory activity has been demonstrated using chemical-induced writhing test and carrageenan paw edema for polyphenolic extract and essential oil of *L. angustifolia* [43]. It is thought that these antioxidant and anti-inflammatory properties are related to the treatment of cancer.

Findings on licorice have shown *in vitro* and *in vivo* anticancer effects [28, 50]. Glycyrrhetinic acid, constituent of *G. glabra* and *G*. *uralensis*, developed anti-initiating and anti-promoting activities [39]. Isoliquiritigenin, one of the flavonoids of *G. glabra*, induced apoptosis of MGC-803 cells through calcium and Deltapsi-dependent pathways [36] as well as cell cycle arrest and cell growth inhibition [32]. Isoliquiritigenin also activated macrophages and cytotoxicity of splenic lymphocytes [33]. Glabrene showed an estrogen receptor-dependent affinity higher than glabridin with growth-promoting effect at low concentration and ER-independent antiproliferative activity at concentrations above 15 mM [37]. Licochalcone-A, phytoestrogen from *G. glabra*, decreased the anti-apoptotic protein bcl-2 and modified the bcl2-/bax ratio in favor of apoptosis [38]. Beta-hydroxy-DHP from *G. Glabra* extract induced Bcl-2 phosphorylation, G2/M cell cycle arrest and apoptosis in breast and prostate tumor cells [34]. Recently, the ethanol extract of *G. uralensis* root has been shown to possess anticancer activity through upregulation of tumor suppressor gene p53, pro-apoptotic protein Bax and p21waf1/cip1 and downregulation of cdk 2 and cyclin E. The extract also caused G1 cell cycle arrest [31]. The antiangiogenic activity of *G. glabra* is also a potential supplemental source to eradicate tumor growth [28].

Other plants have shown interesting results that can suggest their potential uses as antitumoral agents. Essential oils from *L. angustifolia* showed strong antimutagenic activity in *Salmonella typhimurium* strains exposed to the direct mutagen 2-nitrofluorene [41]. Lavender oil and linalool have also been demonstrated to be cytotoxic to human skin cells *in vitro* [42]. Oil from *M. officinalis* induced cytotoxicity on human cancer lines (A549, MCF-7, Caco-2, HL-60, K562) and a mouse cell line B16F10 [49].

Though the herbal remedy tested here has been used extensively in traditional Uighur medicine, its effects on cancer cell growth or metabolism had not been tested previously.

In the present study, we used human hepatoma cells (HepG2) to examine the cytotoxic effects of ASMq ethanol extract to elucidate the initial molecular mechanisms responsible for these effects. We demonstrated that ASMq ethanol extract had significant cytotoxic effect by reducing the cell growth rate and cell viability, increasing the extra-cellular LDH leakage and inhibiting cellular protein, DNA and RNA synthesis.

Inhibiting cancer cell growth has been a continuous effort in cancer treatment. Most anticancer agents act by reducing cell growth and inducing cell death through more or less detailed and complicated pathways. Our results showed that ASMq ethanol extract inhibits HepG2 cell growth in a concentration-dependent manner after 72 hours incubation. Even at a low concentration, there still was a potent inhibitor effect, the 50% inhibition concentration being determined to be about 0.8 mg mL^−1^ in our experimental conditions. This result was confirmed by the neutral red test showing that ASMq ethanol extract decreased HepG2 cell viability in a concentration-dependent manner after 48 hours incubation.

The most frequently used endpoints in cytotoxicity are the breakdown of the cellular permeability barrier, measured by dye exclusion (trypan blue) or by the release of intracellular enzymes like LDH as a consequence of membrane damage or cell detachment [51]. It is well known that cellular injury may lead to a complex sequence of changes to structural and molecular events, frequently culminating in cell death. Many subcellular structures, such as the plasma membrane, nucleus, mitochondria, endoplasmic reticulum and lysosomes are all targets for anticancer agents [52]. The LDH leakage assay is based on the principle that dead cells lose the ability to maintain their plasma membrane integrity, permitting intracellular constituents such as LDH to leak out of the cell into the culture medium. ASMq ethanol extract induced a significant increase in extra-cellular LDH levels at both 48 hours and 72 hours, in a concentration-dependent manner. ASMq ethanol extract also exhibited time-dependent properties on LDH leakage since the effects at 72 hours were greater than at 48 hours. ASMq ethanol extract may alter the integrity of HepG2 cell plasma membranes either directly or as a secondary result of damage to some other cell component and alter the net rates of nutrient uptake in the cells. Therefore, the cytotoxic effects of ASMq ethanol extract might at least in part be correlated with loss of cell plasma membrane integrity and potential.

Lipid peroxidation is one of the consequences of oxidative damage and has been suggested as a general mechanism for cell injury and death. MDA, the end product of lipid peroxidation, has been extensively studied and measured as an index of lipid peroxidation and as a marker of oxidative stress [53]. Many anticancer agents causing cell death are known to induce an overproduction of ROS, and thereby causing oxidative damage to DNA, proteins and lipids and possibly inducing apoptosis. But in our experimental conditions ASMq did not induce significant lipid peroxidation in HepG2 cells after 24 or 48 hours. This indicates that probably the mechanism leading to loss of cell membrane integrity is not due to ROS, but more probably to inhibition of lipoproteins in membrane since as shown above ASMq inhibited protein synthesis.

To explore its effect on cell mechanisms, the effects of ASMq ethanol extract on some basic cell functions such as protein, DNA and RNA synthesis were studied showing a concentration and time-dependent inhibition of DNA and protein synthesis in cells treated with ASMq ethanol extract as reflected by its inhibition of 3H-thymidine and 3H-leucine uptake. Our results showed that ASMq ethanol extract obviously decreased DNA and protein synthesis at 24 and 48 hours incubation. For DNA synthesis, the inhibition was concentration-dependent after 24 and 48 hours incubation, with also a time-dependent effect, the inhibition being greater at 48 than at 24 hours for all concentrations, whereas for protein synthesis the inhibition was maximal from the first concentration at 48 hours. We found dose dependence for RNA synthesis, but no clear time dependence: there was no difference in the inhibition after 24 or 48 hours incubation. DNA, RNA and protein synthesis are directly proportional to cellular growth rate [54, 55]. Our results likely support this viewpoint: the inhibition of cell growth by ASMq ethanol extract seems closely related to cellular DNA, RNA and protein synthesis inhibition. Thus, inhibition of cellular DNA, RNA and protein synthesis might be involved in the mechanisms of ASMq ethanol extract cytotoxicity effect.

## 5. Conclusions

HepG2 cell growth was concentration dependently inhibited by ASMq ethanol extract. Our findings suggest that the cytotoxic effect of ASMq ethanol extract is a combination of its effects in reduction of HepG2 cell growth, alteration of cell membrane integrity and inhibition of cellular protein, DNA and RNA synthesis. As an herbal preparation, ASMq ethanol extract undoubtedly contains a variety of active compounds such as alkaloids, polyphenols or terpenoids, as well as vitamins and fatty acids, which may act on different pathways of cancer cell growth. Further studies will be needed to identify the active compound(s) that confer anticancer activities to ASMq ethanol extract. Once such compounds are identified, the mechanisms by which they exert their effects can begin to be characterized.

## Funding

National Natural Science Foundation of China (30260128); State Administration of Traditional Chinese Medicine of China (2005LHR21).

## Figures and Tables

**Table 1:** Plants contained in Uighur herbal formula: ASMq.

Latin name	Family	Part used	Uighur name	Chinese name
*Adiantum capillus-veneris* L.	Adiantaceae	Whole plant	Pirsiyavxan	Tiexianjue
*Alhagi pseudalhagi* (Bieb.) Desv.	Fabaceae	Branch secretion	Kök tantak	Citang
*Anchusa italica* Retz.	Boraginaceae	Whole plant	Gavziban	Niushecao
*Cordia dichotoma* G.Forst.	Boraginaceae	Fruit	Serbistan	Pobumuguo
*Euphorbia humifusa* Willd.	Euphorbiaceae	Whole plant	Yalmankülak	Dijincao
*Euphorbia maculata* L.				
*Foeniculum vulgare* Mill.	Apiaceae	Fruit	Arpabidiyan	Xiaohuixiang
*Glycyrrhiza uralensis* Fisch. ex DC.	Fabaceae	Radix or rhizoma	Qüqük buya	Gancaogen
*Glycyrrhiza inflata* Batalin	Fabaceae	Radix or rhizoma		
*Glycyrrhiza glabra* L.	Fabaceae	Radix or rhizoma		
*Lavandula angustifolia* Mill.	Lamiaceae	Aerial parts	Üstihuddus	Xunyicao
*Melissa officinalis* L.	Lamiaceae	Whole plant	Badrenjiboye hindi	Mifenghua
*Ziziphus jujuba* Mill.	Rhamnaceae	Fruit	Qilan	Dazao

**Table 2:** Pharmacological activities of plants from Uighur formula: ASMq.

Plant	Uses	Pharmacological properties	Reference
Bugloss	Edible in Mediterranean diet	Radical scavenging, inhibition of H2O2 and Fe2+-chelating activity *in vitro*	[22]
	Stomach ulcer in Middle East	Protective effect on ethanol-induced gastric ulcer in animals (root aqueous extract)	[23]

Common fennel	Medicinal and aromatic herb	Antioxidant activity of methanolic extract	[24]
	Edible in Mediterranean diet	Radical scavenging and iron-chelating activity *in vitro*	[22]
	Edible in Indian diet	Antioxidant activity of aqueous extract (comparison with ascorbic acid)	[25]
	Digestive medicine	Total antioxidant, radical scavenging and metal chelating activities of aqueous and ethanol extracts	[26]

Fragrant manjack	Edible in India	Nutritional value: high level of phosphorus	[22]

Jujube	Aid digestion in China	Immunological activities: induced a rat spleen cells proliferation	[27]

Licorice	Stomachic and cough medicine	*in vitro* and in vivo inhibition of Ehrlich ascites tumor cells proliferation (*G. glabra*)	[28]
		Inhibition of cell proliferation in the MCF-7 breast cancer cell line (*G. uralensis* ethanol extract)	[29]
		Inhibition of H2O2-induced apoptosis of lung fibroblastV79-4 cells (*G. uralensis* methanol extract)	[30]
		Antinephritis activity of licochalcone A (*G. inflata*)	[31]
		Prostate cancer cell growth inhibition of isoliquiritigenin	[32]
		Suppression of pulmonary metastasis by isoliquiritigenin	[33]
		Antitumoral effects similar to those of antimicrotubule agents of beta-hydroxy-DHP (*G. glabra*)	[34]
		Inhibition of lipid peroxidation (*G. glabra*)	[35]
		Isoliquiritigenin induced apoptosis in human gastric cancer MGC-803 cells	[36]
		Biphasic effect on the growth of breast tumor cell of glabridin and glabrene	[37]
		Licochalcone-A from *G. glabra* induced aptotosis of MCF-7 and HL-60 cell lines	[38]
		Protect DNA damage and decrease the stimulation of DNA repair synthesis by glycyrrhetinic acid	[39]

Lavender	Folk medicine	Antioxidant activity (DPPH) of water extract	[40]
	Antiseptic uses	Strong antimutagenic activity of essential oil (bacterial reverse mutation assay)	[41]
	Wound healing	Cytotoxicity of essential oil to human skin cells (*in vitro*)	[42]
	Iranian traditional medicine	Anti-inflammatory activity of polyphenolic fraction and essential oil	[43]
	Turkish folk medicine	Neuroprotective effect against glutamate toxicity (aqueous extract)	[44]
	Inflammatory diseases	Inhibition of lipid peroxidation (phenolic compounds)	[45]

Lemon Balm	Sedative, digestive medicine	Antioxidant of the polar fraction (ethanolic extract, decoction)	[40]
	Folk medicine	Antioxidant capacity (FRAP, DPPH, ABTS) of infusion	[46]
	Mediterranean diet	Antioxidant activity (DPPH) of aqueous extract	[47]
	Bulgarian folk medicine	Antioxidant activity (APTS) of infusion	[48]
	Herbal medicine	Oil cytotoxicity on cell lines A549, MCF-7, Caco-2, HL-60, K562 and B16F10	[49]
	Inflammatory diseases	Inhibition of lipid peroxidation (phenolic compounds)	[45]
